# Assessing the quality of electronic medical records as a platform for resident education

**DOI:** 10.1186/s12909-021-03011-0

**Published:** 2021-11-13

**Authors:** Hsuan Hung, Ling-Ling Kueh, Chin-Chung Tseng, Han-Wei Huang, Shu-Yen Wang, Yu-Ning Hu, Pao-Yen Lin, Jiun-Ling Wang, Po-Fan Chen, Ching-Chuan Liu, Jun-Neng Roan

**Affiliations:** 1 Tainan Municipal North District Kaiyuan Elementary School, Tainan, Taiwan; 2 Institute of Education, National Cheng Kung University, Tainan, Taiwan; 3 Division of Nephrology, Department of Internal Medicine, National Cheng Kung University Hospital Dou-Liou Branch, College of Medicine, National Cheng Kung University, Yunlin, Taiwan; 4 Department of Neurology, National Cheng Kung University Hospital, College of Medicine, National Cheng Kung University, Tainan, Taiwan; 5 Quality Center, National Cheng Kung University Hospital, College of Health Sciences, Chang Jung Christian University, Tainan, Taiwan; 6 Division of Cardiovascular Surgery, Department of Surgery, National Cheng Kung University Hospital, College of Medicine, National Cheng Kung University, Tainan, Taiwan; 7 Department of Internal Medicine, National Cheng Kung University Hospital, College of Medicine, National Cheng Kung University, Tainan, Taiwan; 8 Department of Obstetrics and Gynecology, National Cheng Kung University Hospital, College of Medicine, National Cheng Kung University, Tainan, Taiwan; 9 Department of Pediatrics, National Cheng Kung University Hospital, College of Medicine, National Cheng Kung University, Tainan, Taiwan; 10 Medical Device Innovation Center, National Cheng Kung University Hospital, College of Medicine, National Cheng Kung University, Tainan, Taiwan; 11 Institute of Clinical Medicine, National Cheng Kung University Hospital, College of Medicine, National Cheng Kung University, Tainan, Taiwan

**Keywords:** Note quality, Quality improvement, Resident education

## Abstract

**Background:**

Previous studies have assessed note quality and the use of electronic medical record (EMR) as a part of medical training. However, a generalized and user-friendly note quality assessment tool is required for quick clinical assessment. We held a medical record writing competition and developed a checklist for assessing the note quality of participants’ medical records. Using the checklist, this study aims to explore note quality between residents of different specialties and offer pedagogical implications.

**Methods:**

The authors created an inpatient checklist that examined fundamental EMR requirements through six note types and twenty items. A total of 149 records created by residents from 32 departments/stations were randomly selected. Seven senior physicians rated the EMRs using a checklist. Medical records were grouped as general medicine, surgery, paediatric, obstetrics and gynaecology, and other departments. The overall and group performances were analysed using analysis of variance (ANOVA).

**Results:**

Overall performance was rated as fair to good. Regarding the six note types, discharge notes (0.81) gained the highest scores, followed by admission notes (0.79), problem list (0.73), overall performance (0.73), progress notes (0.71), and weekly summaries (0.66). Among the five groups, other departments (80.20) had the highest total score, followed by obstetrics and gynaecology (78.02), paediatrics (77.47), general medicine (75.58), and surgery (73.92).

**Conclusions:**

This study suggested that duplication in medical notes and the documentation abilities of residents affect the quality of medical records in different departments. Further research is required to apply the insights obtained in this study to improve the quality of notes and, thereby, the effectiveness of resident training.

**Supplementary Information:**

The online version contains supplementary material available at 10.1186/s12909-021-03011-0.

## Introduction

Effective documentation is a key component of medical training. At teaching hospitals, residents are responsible for most of the medical documentation, including inpatient records. They must be able to produce compendious documentation as a core competency for entering residency [1]. An electronic medical record (EMR) includes admission notes, progress notes, weekly summaries, and discharge notes. EMRs form a critical part of e-health systems and are highly instrumental in improving the quality and efficiency of healthcare services; they also serve as legal records [2]. Residents need to learn ways to conduct documentation using the EMR system efficiently while minimising errors.

We consider that the weaknesses in residents’ medical record writing include (1) The problem list affects patient care and health insurance declaration. It is the most important part of medical record writing that needs improvement. Residents usually only write about one disease, which results in treatment problems. (2) The diagnostic plan should be in accordance with the rules of confirm, complete and course follow-up, but most residents write it incompletely. (3) The therapeutic plan should include the content of decision-making. (4) The educational plan should include the preventive measures plan, with the current situation informing the treatment plan. (5) The content of the progress note is always “copied and pasted” from the previous course of the disease without modification, thus is unable to present the authentic course of the disease.

We reviewed previous studies to assess note quality and use of EMR as part of medical training [3–10]. However, we found these studies had limitations for assessing our residents’ medical record writing. For example, Baker et al [3] developed an interpretive summary, differential diagnosis, explanation of reasoning, and alternatives assessment tool to assess comprehensive new patient admission notes. Liang et al [8] created a checklist for neurology residents” documentation. These tools are used for a specific section in medical records or a neurology department, which may not be practical for other specialties. Thammasitboon et al [4] created the Assessment of Reasoning Tool that allows teachers to assess clinical reasoning and structure feedback conversations, which is an incomplete fit for assessing writing. Lee et al [5] investigated the use of medical record templates in an EMR system, and the result showed that residents appreciate templates, but the overall documentation quality remained unknown. Other studies have developed curricula for better understanding of medical documentation [6, 7, 9, 10]. However, these curricula and study results are insufficient for use with medical record writing assessment.

In this regard, a clinically-applicable generalized note quality assessment tool needs to be developed. Our residents faced challenges in using the EMR systems for documentation in daily clinical practice. For example, the transition from clinical observation to medical writing and then to the EMR system requires time and effort. In the current EMR system, information is often presented in segments, splitting data from multiple screens and modules in various formats. EMR writing could overwhelm residents with massive volumes of data; it may stunt critical clinical thinking and restrict doctor-patient communication, weakening interprofessional collaboration [11–15]. Consequently, using note templates in the EMR system and copy-and-paste from previous notes are frequently used strategies; however, these dramatically decrease the quality of medical records [16, 17]. Although some departments have created medical record templates based on their daily routine, the effectiveness of these templates lacks proper evaluation and investigation.

From 2018 to 2019, the Medical Quality Review Committee (MQRC) in our hospital held a competition to improve the note quality of EMRs. The competition aimed to raise healthcare providers’ awareness of medical records and investigate medical record training in departments/stations so that clinical teachers could construct a systematic medical record training programme. Hospital staff, including attending physicians, residents, interns, nurse practitioners, and medical students participated. The competition comprised two stages: the first stage contained (1) medical record quality review (50%) and (2) self-report (20%); the second stage was an oral presentation (30%). The winners received awards and certificates. This competition was funded by our hospital and the MQRC.

This study reports the assessment of note quality obtained by reviewing medical records. It aims to identify the critical issues in the context of EMRs written by residents from various departments. The research questions are as follows: (1) What is the overall note quality among residents of different specialties? (2) Does note quality vary between residents of different departments/specialties? This study was reviewed and approved by the Institutional Review Board of the National Cheng Kung University Hospital.

## Method

### Data collection

Out of 37 departments in our hospital, 32 participated in the competition; the participation rate was 87% (Table 1). The administrative staff of each station—who had no prior knowledge of competition—were asked randomly to select five inpatient records by using a computer-generated list from the stations. Ultimately, 149 inpatient records were collected.

**Table 1:** Number of Medical Records from Each Department

Group	General Medicine	Surgery	Paediatrics	Obstetrics and Gynaecology	Other Departments	Total
Departments/ Stations	Gastroenterology, Geriatrics, Rehabilitation, Nephrology, Cardiology, Oncology, Haematology, Hospice, General Medicine, Rheumatology, Infectious Diseases, Cardiac Intensive Care Unit, General Medicine Intensive Care Unit, Respiratory Care Unit, Neurology, Psychiatry	General Surgery, Thoracic Surgery, Cardiovascular Surgery, Plastic Surgery, Neurosurgery, Surgical Intensive Care Unit, Orthopaedics, Urology	Paediatric Ward, Paediatric Intensive Care Unit, Baby Room	Obstetrics and Gynaecology Ward, Delivery Room	Ears, Nose, and Throat, Ophthalmology, Oral Surgery	
Number	16	8	3	2	3	32
Inpatient records	75	41	14	8	11	149

### Rating process

To examine the quality of inpatient records, the MQRC first produced a draft checklist based on our hospital’s clinical needs and a literature review [3–10]. Considering our needs in assessing overall documentation quality in clinical environments, the MQRC then held interactive workshops involving multiple departments at our hospital. In the workshops, doctors and medical experts considered the unique needs of each specialty. Medical records committee members and senior doctors in the MQRC revised the checklist based on workshop feedback.

The final version of the inpatient record checklist contained six note types and twenty items (Table 2). The six note types were as follows: (1) admission notes (25%); (2) problems list (8%); (3) progress notes (25%); (4) weekly summaries (4%); (5) discharge notes (25%); and (6) overall performance (13%). The total score was 100. Furthermore, an ‘outstanding performance’ fetched an additional nine points (Table 2, Items 21–23). Each note type included a different percentage of points based on their functions in an inpatient record. Each note type and item were rated and scored as excellent, good, fair, or poor.

**Table 2:** Inpatient Record Checklist

Note type	Item	Excellent	Good	Fair	Poor
Admission notes (25%)	1. Present illness is clear, chronologically arranged, and segmented; the records are complete and correct.	8	7	6	0
	2. Positive findings in ‘review of system’ should be specified in detail.	5	4	3	0
	3. Physical examination is consistent with the symptoms of the disease and corresponds to the diagnosis and treatment plan.	7	6	5	0
	4. ‘Education plan’ records prevention programmes rather than treatment plans.	5	4	3	0
Problems list (8%)	5. Daily admission problems are recorded properly and completely.	4	3	2	0
	6. The content of the problems list corresponds to the progress record.	4	3	2	0
Progress notes (25%)	7. The course record does not have a copy-and-paste format or repeated typing errors.	7	6	5	0
	8. Describe the treatment and the symptoms, which are compared to those in the previous days. Record the differences between new and old laboratory data, e.g. the antibiotic treatment days, days after surgery, and radiation therapy.	3	2	1	0
	9. Record new changes in the problem or condition, followed by revised diagnosis and treatment plans.	6	5	4	0
	10. No duplications of objective information, which recorded experimental data, image inspection, etc. in ‘assessments’. Note: important data are allowed to be duplicated in daily records. Serial data should be shown in brief.	3	2	1	0
	11. The plan contains indications for important laboratory tests, ranges of value, image, and other study findings. Also include the corresponding plans to those tests results.	3	2	1	0
	12. The invasive procedure or the surgical procedure is comprehensively recorded (the surgical record containing the intraoperative image, or the plotter is rated as ‘excellent’).	3	2	1	0
Weekly summary (4%)	13. The record is concise and includes admission date, main diagnosis and reasons for admission, procedures during the past 1 week, disease course and responses to treatments, plans for the following week (not copying admission note).	4	3	2	0
Discharge notes (25%)	14. The discharge diagnosis is comprehensive. No informal abbreviations are present. The diagnosis is written in order of importance and includes comorbidities. (If there is any operation record, the pre-operative, post-operative diagnosis and the term of the surgery procedures should be correct and comprehensive).	8	7	6	0
	15. The content of the discharge summary is concise, brief, and logical.	5	4	3	0
	16. Examination results, including laboratory data, images, electrocardiogram, pathology, special examination, etc., are summarised without redundant contents (e.g. a full copy of examination reports with content unrelated to the discharge diagnosis).	4	3	2	0
	17. The discharge summary is revised according to attending physicians’ feedback in admission medical records. Any error in the admission notes should have been corrected by the attending physician and should not re-appear in the discharge notes.	8	7	6	0
Overall performance (13%)	18. Assessment, differential diagnosis, and treatment plans are logical and reasonable.	5	4	3	0
	19. Electronic medical records (EMRs) are written consistently without informal symbols, characters, or abbreviation.	4	3	2	0
	20. The descriptions are clear. No grammatical or spelling errors are present.	4	3	2	0
Extra points (One item gain 3 points)		Yes		No	
Outstanding performance	21. The therapeutic plan includes shared decision-making (SDM) meeting records and/or conference records on treatment plans with patients and families				
	22. There is an evidence-based record, with literature review, for rare cases.				
	23. The results are summarised in a case conference, a combined conference, or a grand round in EMRs.				

The seven raters were from the departments of Nephrology (1), Neurology (1), Internal Medicine (1), Obstetrics and Gynaecology (1), Paediatric (1), and Cardiovascular Surgery (2). To avoid discrepancies caused due to multiple authors, the rating process only used notes written by a single resident. The raters independently scored 149 medical records based on the checklist, and the final scores were averaged.

### Data analysis

This study used the scores obtained for six note types and twenty items. Data were categorised into five groups: general medicine, surgery, paediatrics, obstetrics and gynaecology, and other departments. Scores were analysed using descriptive statistics, and ANOVA was used to examine the average differences between and within groups. Owing to the unequal variance of the five groups, we used generalized least squares instead of ordinary least squares to calculate the differences.

## Results

### Overall performance

The overall quality of medical records was rated from ‘fair’ to ‘good’ (Table 3).

**Table 3:** Descriptive Statistics of Medical Record

Note type	Items	Range	Mean (SD)
Admission notes (25%)	01	0–8	6.99(0.83)
	02	0–5	3.57(0.91)
	03	0–7	5.64(1.39)
	04	0–5	3.47(1.17)
Problem list (8%)	05	0–4	2.92(0.70)
	06	0–4	2.94(0.68)
Progress notes (25%)	07	0–7	5.46(1.52)
	08	0–3	1.87(0.67)
	09	0–6	4.84(0.86)
	10	0–3	1.82(0.61)
	11	0–3	1.84(0.57)
	12	0–3	2.03(0.54)
Weekly summary (4%)	13	0–4	2.69(0.80)
Discharge notes (25%)	14	0–8	6.81(1.17)
	15	0–5	3.90(0.64)
	16	0–4	2.68(0.67)
	17	0–8	6.88(0.70)
Overall performance (13%)	18	0–5	3.88(0.60)
	19	0–4	2.80(0.71)
	20	0–4	2.82(0.60)

### Inter-comparison among note types and the five groups

To investigate each note type’s performance, we calculated the standardised scores (group mean/total score). As Table 4 shows, the weekly summary needed the most improvement (0.66), followed by progress notes (0.71), problem list (0.73), overall performance (0.73), admission notes (0.79), and discharge notes (0.81) (Table 4). The format of progress notes was the subjective-objective-assessment-plan (SOAP), but we found that instances of duplication often occurred in the ‘subjective’ section, which led to low scores in this note type. This finding showed that residents used a copy-and-paste strategy rather than revising patients’ daily changes and summarising daily information when writing progress notes.

**Table 4:** Means, Standard Deviation (SD), and Standardised Scores (Std.)

	General Medicine		Surgery		Paediatrics		Obstetrics and Gynaecology		Other Departments		Overall Mean	
	Mean (SD)	Std. score	Mean (SD)	Std. score	Mean (SD)	Std. score	Mean (SD)	Std. score	Mean (SD)	Std. score	Mean (SD)	Std. score
**Admission notes** (25%)	19.75 (2.98)	**0.79**	18.91 (3.66)	**0.76**	20.14 (2.28)	**0.81**	19.94 (1.99)	**0.80**	21.09 (1.99)	**0.84**	19.68 (3.06)	**0.79**
**Problems list** (8%)	5.84 (1.36)	**0.73**	5.64 (1.34)	**0.71**	6.09 (1.28)	**0.76**	6.00 (0.93)	**0.75**	6.41 (1.14)	**0.80**	5.87 (1.32)	**0.73**
**Progress notes** (25%)	17.54 (3.59)	**0.70**	17.79 (4.03)	**0.71**	18.52 (2.77)	**0.74**	18.29 (2.53)	**0.73**	18.74 (3.62)	**0.75**	17.86 (3.61)	**0.71**
**Weekly summary** (4%)	2.75 (0.67)	**0.69**	2.48 (1.04)	**0.62**	2.74 (0.72)	**0.69**	2.98 (0.58)	**0.75**	2.77 (0.67)	**0.69**	2.69 (0.80)	**0.66**
**Discharge notes** (25%)	20.32 (2.31)	**0.81**	19.83 (2.92)	**0.79**	20.41 (1.64)	**0.82**	20.75 (1.23)	**0.83**	20.95 (2.01)	**0.84**	20.27 (2.38)	**0.81**
**Overall performance** (13%)	9.40 (1.66)	**0.72**	9.25 (1.74)	**0.71**	9.77 (1.23)	**0.75**	10.06 (0.94)	**0.77**	10.23 (1.52)	**0.79**	9.51 (1.62)	**0.73**
**Total** **(100%)**	75.58 (10.29)		73.92 (10.61)		77.67 (8.23)		78.02 (5.86)		80.20 (7.62)		75.87 (9.91)	

Additionally, we compared the total score differences between the five groups. The EMRs conducted by other departments gained the highest total score (80.20), followed by obstetrics and gynaecology (78.02), paediatrics (77.47), general medicine (75.58), and surgery (73.92).

To understand the performance of each item among the five groups, ANOVA analysis was conducted with grand means as a reference group. As Table 5 shows, residents of other departments gained significantly higher scores than the grand mean in the following note types:

1. Admission notes: Item 3 (0.76) and Item 4 (0.36)
2. Problem list: Item 5 (0.24) and Item 6 (0.30)
3. Progress notes: Item 8 (0.17) and Item 12 (0.40)
4. Weekly summary: none
5. Discharge notes: Item 14 (0.40) and Item 15 (0.18)
6. Overall performance: Items 18 (0.15), 19 (0.32), and 20 (0.26)

**Table 5:** ANOVA Analysis among Five Groups with Grand Mean as Reference Group

Note type	Items	General Medicine	Surgery	Paediatrics	Obstetrics and Gynaecology	Other Departments
		Coefficient (SE)^a^.	Coefficient (SE).	Coefficient (SE).	Coefficient (SE).	Coefficient (SE).
Admission notes	01	0.02 (0.05)	−0.18* (0.01)	0.14 (0.08)	0.24* (0.09)	0.13 (0.11)
	02	0.00 (0.06)	− 0.13 (0.07)	0.23* (0.09)	− 0.03 (0.13)	0.16 (0.11)
	03	− 0.05 (0.08)	− 0.26* (0.13)	0.28* (0.12)	0.05 (0.18)	0.76* (0.09)
	04	0.10 (0.07)	− 0.21* (0.09)	− 0.19 (0.14)	0.01 (0.17)	0.36* (0.14)
Problems list	05	0.01 (0.04)	−0.13* (0.05)	0.10 (0.07)	0.04 (0.10)	0.24* (0.08)
	06	−0.04 (0.04)	−0.09 (0.05)	0.13 (0.08)	0.10 (0.10)	0.30* (0.07)
Progress notes	07	−0.03 (0.09)	−0.18 (0.11)	0.29* (0.14)	0.23 (0.18)	0.24 (0.16)
	08	−0.06 (0.04)	0.02 (0.05)	0.10 (0.08)	−0.03 (0.10)	0.17* (0.09)
	09	0.01 (0.05)	−0.06 (0.07)	0.16* (0.07)	−0.03 (0.13)	−0.03 (0.12)
	10	−0.03 (0.04)	0.00 (0.05)	0.09 (0.06)	0.06 (0.08)	0.03 (0.07)
	11	−0.02 (0.04)	−0.02 (0.04)	0.11 (0.06)	−0.03 (0.08)	0.09 (0.07)
	12	−0.18* (0.03)	0.17* (0.04)	−0.08 (0.05)	0.22* (0.09)	0.40* (0.10)
Weekly summary	13	0.06 (0.05)	−0.21* (0.06)	0.05 (0.08)	0.29* (0.08)	0.08 (0.09)
Discharge notes	14	0.06 (0.06)	−0.32* (0.13)	0.11 (0.10)	0.25 (0.11)	0.40* (0.15)
	15	−0.02 (0.04)	−0.04 (0.05)	− 0.01 (0.07)	0.12 (0.09)	0.18* (0.08)
	16	0.02 (0.04)	−0.06 (0.06)	−0.02 (0.08)	0.03 (0.09)	0.09 (0.07)
	17	−0.02 (0.05)	−0.02 (0.06)	0.06 (0.06)	0.08 (0.06)	0.01 (0.08)
Overall performance	18	−0.02 (0.04)	−0.06 (0.05)	0.07 (0.06)	0.15* (0.07)	0.15* (0.08)
	19	−0.02 (0.04)	−0.15* (0.06)	0.14* (0.07)	0.22* (0.08)	0.32* (0.07)
	20	−0.06 (0.04)	−0.04 (0.05)	0.06 (0.06)	0.18* (0.07)	0.26* (0.06)

*p < 0.05

^a^*SE* Standard Error

Among the twenty items, surgical residents gained only one item score higher than the grand mean (Item 12 in progress note, 0.17). In addition, surgical residents showed significantly low scores on the following seven items:

1. Admission notes: Items 1 (− 0.18), 3 (− 0.26), and 4 (− 0.21).
2. Problem list: Item 5 (− 0.13).
3. Weekly summary: Item 13 (− 0.21).
4. Discharge notes: Item 14 (− 0.32).
5. Overall performance: Item 19 (− 0.15).

For general medicine, only the Item 12 (− 0.18) score was significantly lower than the grand mean. Paediatric residents received scores significantly higher than the grand mean for Items 2 (0.23), 3 (0.28), 7 (0.29), 9 (0.16), and 19 (0.14). Obstetrics and gynaecology residents earned scores significantly higher than the grand mean for Items 1 (0.24), 12 (0.22), 13 (0.29), 18 (0.15), 19 (0.22), and 20 (0.18).

## Discussion

This study contributes to the assessment of note quality in residency in the EMR era. Based on previous studies that assessed note quality in a particular format or a specific department [3, 4, 8], we developed an overall clinically usable checklist and assessed its effectiveness by examining our residents’ medical record writing. The results indicated that residents from different departments demonstrated diverse levels of performance. This finding expanded previous studies indicating that residents demonstrated inconsistencies in note quality [5, 15, 18, 19]. This finding gives new insight into note quality and can be useful for creating medical record templates. The results also suggest that duplications and incorrect documentation processes obscure the use of the EMR system in complex clinical scenarios.

First, of the six note types, the weakest was the weekly summary, followed by progress notes. Subsequently, we found that some residents copied data from admission notes to create progress notes instead of renewing daily examination results. As EMR systems comprise large volumes of data, residents need to record everyday changes and select useful data to complete precise and concise documentation. Unlike discharge notes—which show a summary with written orders—weekly summaries and progress notes required careful clinical reasoning skills, including collecting the patient’s background details and screening data to assess relevant and critical information. The massive amount of data in the EMR system may hinder the process of constructing the notes de novo or receiving essential feedback from residents’ advisers or colleagues. Residents might ignore the inter-connection among note types within EMRs, which leads to a long but pointless record. In addition, when residents relied solely on copy-and-paste from previous records or templates in the EMR system, the feedback from senior doctors was likely not meaningful because the records were created through computer programming rather than critical thinking. In the United States, more than 60% of residents duplicate data without confirming their accuracy, which leads to 2–3% of inappropriate diagnoses [20]. Hence, the competencies of medical knowledge and system-based practice could be particularly vital for improving note quality in the EMR system [11, 20].

Second, we found differences among the groups. Performance varied depending on the unique affordances and workloads of each department. Surgery had the lowest score among the five groups. They performed well solely on Item 12, which was the record for the operation. This could be a result of the specific culture of surgery, for example, heavy workload, time pressure, insufficient information from interviews, lack of feedback, and the focus on surgical skills training. As such, we propose that surgical residents might require extra opportunities to learn clinical reasoning processes and chronological descriptions of the EMR system, rather than relying overly on templates and the copy-and-paste strategy.

Conclusively, meaningful experiences in using the EMR system should be consistently implemented in clinical training. The integration of the EMR system and core competencies should be rigorously designed and assessed. Enhancing EMR as a useful tool could benefit not only the level of clinical skills of residents but also their attitudes and professionalism in clinical practice [12, 21].

### Pedagogical suggestion

A previous study suggested that EMRs can influence the ways in which residents develop clinical reasoning skills and document strategies [21]. We further suggest that eliminating duplication should be a key factor in resident education. The findings of this study show that our residents may lack a comprehensive review of the systems and patients’ clinical information. In the era of specialised divisions in medicine, a comprehensive review is necessary because it may affect more than 10% of final clinical diagnoses [22]. Some suggestions are provided to mitigate the negative educational impact of EMRs. First, meetings for residents, attending physicians, and other healthcare providers can be held for effective interprofessional interactions. Second, residents can be trained to interact with patients before directly reviewing EMRs [17] to decrease their reliance on computer data. Third, the introduction of a systematic coaching programme that integrates important elements and strategies as a comprehensive course can help clinical teachers train residents effectively.

### Limitations

This study had some limitations. First, an inpatient record checklist was created for the clinical assessment. The development process largely relied on the discussions of medical experts. Lack of validity is a major limitation, and the checklist needs further revision. Second, although the sample comprised residents from various departments, it only included 7–10% of the residents in our hospital. In future studies, we will expand the sample size to other hospitals and examine the checklist’s reliability and validity to promote generalization. Despite these limitations, this study provides new insights into the directions for future studies on residents’ note quality and the use of EMRs.

## Conclusion

This study adds to the existing body of literature and demonstrates the need to improve note quality in residents’ EMRs. In addition, the various results of note types and groups offer potential insights into the diverse cultures and needs of EMR training for residents in different specialties. From the assessment of authentic medical records in the EMR system, we provided pedagogical suggestions that thoroughly encompassed the spectrum of resident education.

## Supplementary Information

**Additional file 1: Supplemental file** A. Note template 1. For intensive care unit patients. B. Note template 2. For intensive care unit patients. C. Note template for endocrinology patients.

## Data Availability

Not applicable.

## Abbreviations

ANOVA: Analysis of variance
EMR: Electronic Medical Record
MQRC: Medical Quality Review Committee
SD: Standard Deviation
SE: Standard Error
Std.: Standardised Scores
