# Supplementary material for: Assessing the quality of electronic medical records as a platform for resident education
Source: BMC Med Educ. 2021 Nov 13;21:577. doi: 10.1186/s12909-021-03011-0 (PMC8590775; doi:10.1186/s12909-021-03011-0)
Supplement: Supplementary file 1 — Additional file 1: Supplemental file A. Note template 1. For intensive care unit patients. B. Note template 2. For intensive care unit patients. C. Note template for endocrinology patients. [file 12909_2021_3011_MOESM1_ESM.doc]

**Supplemental file**

**A. Note template 1. for intensive care unit patients**

■Chief Complaints：

Fever, chills, and vomiting on YYYY/MM/DD.

■Present Illness：

The 62-year-old woman has DM, CAD, Af, PAOD, ESRD on HD, high degree AV block s/p PPM,

and hiatal hernia. She lives at home and needs assistance to perform activities of daily living. She could ambulate with a walker.

She had fever, chills, and vomiting on YYYY-MM-DD. Other symptoms included dyspnea, possible runny nose, no sore throat, headache, abdominal pain, diarrhoea, dysuria, or myalgia. She was brought to the NCKUH emergency department (ED) on YYYY/MM/DD early morning.

Fever with hypoxemia was documented during triage. Hyperkalaemia was soon revealed; therefore, she received medical therapy and daily haemodialysis (HD) during the ED stay (DATE01-DATE04). She had lung infiltrates on CXR, and pneumonia was suspected. Empiric ampicillin-sulbactam was administered to treat febrile illness. She needed a Venturi mask during DATE01-DATE03 due to hypoxemia. Her dyspnea worsened; therefore, non-invasive ventilation was applied on YYYY/MM/DD early morning. Chest CT showed bilateral upper and lower lung consolidations, confirming pneumonia. She was then admitted to the medical ICU on YYYY/MM/DD evening after HD.

■Past History：

- End-stage renal disease on haemodialysis tiw: Dry weight ~ 85 kg
- Type 2 diabetes mellitus: glimepiride 2mg bidac
- Coronary artery disease: A coronary angiogram was suggested, but the patient and her family would like to have more discussions with the cardiologist.
- Peripheral arterial occlusive disease/p percutaneous transluminal angioplasty for left superficial femoral artery to popliteal artery (YYYY-MM)
- Cirrhosis, Child-Pugh A, HCV related
- Renal hyperparathyroidism s/p total parathyroidectomy and auto transplantation in 2016-04
- Hiatal hernia
- Paroxysmal atrial fibrillation
- High-degree atrioventricular block with bradycardia/p permanent pacemaker (DDD) on September 2016/09/05
- TOCC: Travel history (-), occupation (-), contact history (-), cluster (-), not within her family at least (January 2018/01/27)

■Physical Examination：

-Vital Signs: (Automatically fill in)

-Appearance: lethargic

-Consciousness: E4V5M6

-Pupils: 2/2, LR -/-

-Conjunctivae: not pale Sclera: anicteric

-HEENT: No conjunctival injection

-Chest wall: no lesions

-Heart: regular heart beats, small heart sounds, no murmur

-Lungs: bilateral lower lung crackles

Abdominal: distended, normoactive bowel sounds, no tenderness

-Inguinal area: no lesions

-Extremities: no oedema, cold at the toes

-Skin: some abrasions in the bilateral lower limbs

**B. Note template 2. for intensive care unit patients**

■Chief Complaints：

Loss of consciousness / cardiac arrest on YYYY/MM/DD evening

■Present Illness：

A 60-year-old woman had diabetes mellitus and heart failure with preserved ejection fraction (HFpEF), HTN, dyslipidaemia, hypothyroidism, and morbid obesity with obesity hypoventilation syndrome. She lives at home and is cared for by her son. She could ambulate to the toilet at baseline but became bedridden for two weeks before admission due to weakness. Bilateral leg limb erythema had been noted for more than one month.

She had a cough and poor intake for five days with progressive drowsiness. She did not report any other discomfort. She remained interactive on YYYY/MM/DD noon but was found unresponsive on YYYY/MM/DD evening. When the EMT arrived, cardiac arrest was revealed; therefore, she received CPR and was sent to the NCKUH emergency department (ED).

In the ED, the initial rhythm was asystole. ROSC was achieved after CPR for 13 minutes. ECG showed no ST elevation, and bedside transthoracic echocardiogram showed no systolic dysfunction or regional wall motion abnormality. The cardiologist considered coronary angiography to be not beneficial. The survey showed leukocytosis with left shift, extremely high CRP (362 mg/L), and acute kidney injury (Cr 1.07 → 2.95 mg/dL). Right lung opacities were demonstrated on CXR. Dopamine was administered to treat hypotension. She was then admitted to the medical ICU at YYYY/MM/DD night.

■Past History：

- Diabetes mellitus

HbA1C YYYY-MM: 9.1

Rx: Novomix 30U bidac

- Heart failure with preserved LV ejection fraction Rx: Isosorbide MN 60 mg qd
- Morbid obesity with obesity hypoventilation syndrome BW YYYY: 88–96kg
- Hypertension

Rx: doxazosin 4 mg bid, nifedipine OROS 30 mg bid, irbesartan 150mg bid

- Dyslipidaemia, no Rx.
- Hypothyroidism

Rx: thyroxine-L 100 mc/day

- TOCC: Travel history (-), occupation (-), contact history (-), cluster (-) (2018/02/03)

■Physical Examination：

-Appearance: obese, chronic ill-looking, oddor+

-BW 116 kg

-Consciousness: E1VeM3 (ventilated via an endotracheal tube)

-Vital signs: T= 35.1°C, P=89, R=20, BP=108/68 mmHg

-Pulpils: 2.5/2.5, LR -/-

-Conjunctivae: not pale Sclera: anicteric

-HEENT: no visible lesion, too overweight for deep palpation

-Chest wall: no visible lesions

-Heart: regular heartbeats, no murmur

-Lungs: Coarse breath sounds in the right lung field

Abdominal: flat, hypoactive bowel sounds, no tenderness

-Inguinal area: no palpable lesions

-Extremities: bilateral lower limb swelling and erythema with superficial wounds

-Skin: superficial wounds in the bilateral lower limbs and buttocks


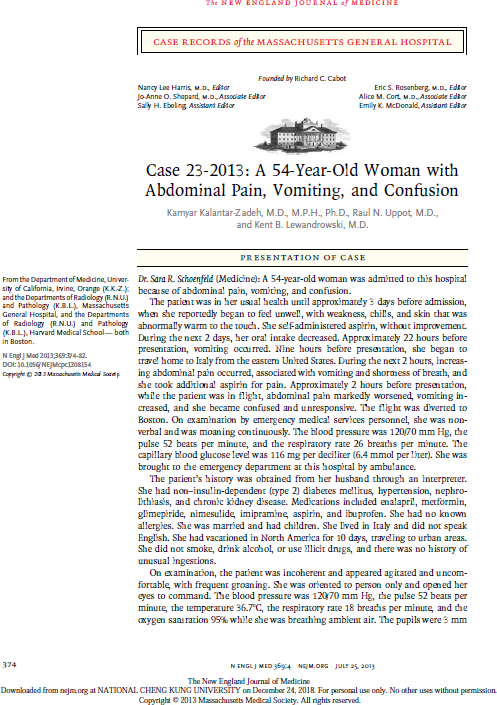
**C. Note template for endocrinology patients**

Case: A 54-Year-Old Woman with Abdominal Pain, Vomiting, and Confusion (N Engl J Med 2013; 369:374-382) <https://www.nejm.org/doi/pdf/10.1056/nejmcpc1208154>

Diagnosis:

**Type B lactic acidosis caused by metformin accumulation.**

Briefly describe chief complaint.

A 54-year-old woman was admitted to this hospital because of

abdominal pain, vomiting, and confusion.

The patient was in her usual health until approximately three days before admission, when she reportedly began to feel unwell, with weakness, chills, and skin that was abnormally warm to the touch. She self-administered aspirin without improvement. During the next two days, her oral intake decreased. Approximately 22 hours before presentation, vomiting occurred. Nine hours before presentation, she began to travel home to Italy from the eastern United States. During the next two hours, increasing abdominal pain occurred, associated with vomiting and shortness of breath, and she took additional aspirin to alleviate pain. Approximately two hours before presentation, while the patient was in flight, abdominal pain markedly worsened, vomiting increased, and she became confused and unresponsive. The flight was diverted to Boston. Upon examination by emergency medical services personnel, she was nonverbal and was moaning continuously. The blood pressure was 120/70 mm Hg, the pulse 52 beats per minute, and the respiratory rate 26 breaths per minute. The capillary blood glucose level was 116 mg per decilitre (6.4 mmol per litre). She was brought to the emergency department at this hospital by ambulance.

Present illness

1. Writing in chronological order.
2. The symptom of pain should indicate the location, the nature, the severity, the duration, and whether there are predisposing factors or alleviating factors.
3. In cases of hyperglycaemia emergency or insufficient blood sugar control, there should be an assessment of precipitating factors (5I) in the present illness, indicating whether there is infection, steroid use, alcohol consumption, heart disease or stroke, or related symptoms of ischemia bowel and whether the medication is regular. These are important negative findings.

Past history should be considered in present illness if it is helpful to the differential diagnosis. Otherwise, it should be listed in the section of past history.

If the medication record is helpful for the differential diagnosis, it should be considered in present illness, preferably with the dose and frequency.

Personal history should be considered in present illness if it is helpful for differential diagnosis.

The patient’s history was obtained from her husband through an

interpreter. She had non-insulin-dependent (type 2) diabetes mellitus, hypertension, nephrolithiasis, and chronic kidney disease. Medications included enalapril, metformin, glimepiride, nimesulide, imipramine, aspirin, and ibuprofen. She had no known

allergies. She was married and had children. She lived in Italy and did not speak English. She had vacationed in North America for 10 days, travelling to urban areas. She did not smoke, drink alcohol, or use illicit drugs, and there was no history of unusual ingestions.

Upon examination, the patient was incoherent and appeared agitated and uncomfortable, with frequent groaning. She was oriented to person only and opened her eyes to command. The blood pressure was 120/70 mm Hg, the pulse 52 beats per minute, the temperature 36.7°C, the respiratory rate 18 breaths per minute, and the oxygen saturation 95% while she was breathing ambient air. The pupils were 3 mm in diameter and minimally reactive to light; the oral mucous membranes were dry, and the neck was supple. The abdomen was soft, without distention, rebound tenderness, or guarding. The skin was cool. The remainder of the general examination was normal. The neurologic examination was limited because of the patient’s inability to follow commands; she withdrew all extremities to pain, and cranial nerves and strength appeared normal. Normal saline was rapidly infused, and dextrose, insulin, ondansetron, and morphine sulphate were administered intravenously. An electrocardiogram revealed atrial fibrillation at a rate of 115 beats per minute and a QRS duration of 94 msec, with a tremulous baseline possibly obscuring ST-segment depression in the inferior leads. Blood levels of calcium, triglycerides, glycated haemoglobin, and haptoglobin were normal, as were the results of liver-function tests; other test results are shown in [Table 1](https://www-nejm-org.er.lib.ncku.edu.tw/doi/10.1056/NEJMcpc1208154). Placement of an indwelling urinary catheter was followed by that of intravascular catheters in the right external jugular vein and the femoral artery.

1. Units should be added to vital signs.
2. Physical examination: The differential diagnosis related to the important positive and negative findings should be recorded. The marks such as (+), (-), nil, NP, or unremarkable should be avoided in physical examination.
3. Laboratory data briefly describe the test results related to the chief complaint and marks the unit.

Within two hours after the patient’s arrival in the emergency department, tachypnoea and increasing somnolence developed; the results of venous oximetry are shown in [Table 1](https://www-nejm-org.er.lib.ncku.edu.tw/doi/10.1056/NEJMcpc1208154). The trachea was intubated after the administration of etomidate and rocuronium, 100% oxygen was administered, and bicarbonate was infused. A chest radiograph showed no evidence of pneumonia or pleural effusion. There were ill-defined calcifications in the soft tissue of the left breast.

Approximately three hours after the patient’s arrival, the rectal temperature decreased to 31.7°C and the blood pressure to 84/43 mm Hg. Norepinephrine bitartrate and bicarbonate were administered; fluids were warmed before infusion, and a blanket warmer was placed. Dark-brown gastric secretions that were positive for occult blood were aspirated through an orogastric tube; the gastric pH was 5.7.

Computed tomography (CT) of the abdomen and pelvis without the administration of intravenous or oral contrast material ([Figure 1](https://www-nejm-org.er.lib.ncku.edu.tw/doi/10.1056/NEJMcpc1208154)) revealed pancreatic oedema, peripancreatic fat stranding, a small amount of perihepatic and pericholecystic fluid without biliary ductal dilatation, some thickened walls in several loops of small bowel, and an atrophic left kidney containing a non-obstructing calculus. CT of the chest revealed dependent atelectasis, with no focal consolidation, masses, or effusions, and calcifications of the left breast. CT of the brain was normal.

Cefepime, vancomycin, and metronidazole were administered intravenously. After laboratory results were known, sodium polystyrene sulfonate was given orally. Toxicologic screening of the blood and urine was negative.

The patient was admitted to the cardiac intensive care unit (ICU). Vasopressin, propofol, and calcium were added, and additional bicarbonate and glucose were administered. Eight hours after her presentation, continuous venovenous hemofiltration with bicarbonate solution was begun. Cultures of the blood and urine were obtained. Fourteen hours after presentation, the urine sodium level was 136 mmol per litre, and the urine creatinine level was 0.25 mg per millilitre. Echocardiography revealed normal global cardiac function, without pericardial effusion.

Hospitalisation/Inpatient records

1. Record important events chronologically.
2. The record should indicate whether the acute problem has been completely resolved.
3. Avoid lengthy narratives.

Ultrasonography of the abdomen revealed small-volume ascites, nonspecific thickening of the gallbladder wall, and an atrophic left kidney; there was increased renal parenchymal echogenicity of both kidneys ([Figure 2](https://www-nejm-org.er.lib.ncku.edu.tw/doi/10.1056/NEJMcpc1208154)).

During the first 17 hours, the patient had oliguria, with approximately 125 ml of urine excreted.

Within the first 24 hours after admission, her mental status improved dramatically. She was extubated one day after admission. **Within the next 48 hours, her metabolic abnormalities started to normalise, and she began to make copious amounts of urine**. At that point, continuous venovenous hemofiltration was discontinued, and she was weaned off vasopressin. Out of concern for infection, she had been started on broad-spectrum antibiotics at the time of admission. However, after 48 hours, cultures of blood and urine remained negative, and the antibiotics were discontinued. She was transferred to the general medical unit, where her hypertension was managed with a calcium-channel blocker. Her renal function completely normalised. She was discharged from the hospital one week after admission, and she returned home to Italy.
